# Application of Calcium Sulfate Whiskers to Cement-Based Materials: A Review

**DOI:** 10.3390/ma17051138

**Published:** 2024-02-29

**Authors:** Guoqiang Liu, Yongpang Liao, Xun Sha, Guangmin Liu, Yingjie Zhang, Rongxin Guo, Yao Yue

**Affiliations:** 1 Faculty of Metallurgical and Energy Engineering, Kunming University of Science and Technology, Kunming 650093, China; liuguoqiangkust@yeah.net (G.L.); zyjkmust@126.com (Y.Z.); 2 Faculty of Civil Engineering and Mechanics, Kunming University of Science and Technology, Kunming 650500, China; liaoyongpang@stu.kust.edu.cn (Y.L.); shaxun@stu.kust.edu.cn (X.S.); liuguangmin888@stu.kust.edu.cn (G.L.); guorx@kust.edu.cn (R.G.); 3 Yunnan Key Laboratory of Disaster Reduction in Civil Engineering, Kunming 650500, China; 4 International Joint Laboratory for Green Construction and Intelligent Maintenance of Yunnan Province, Kunming 650500, China; 5 Department of Highway and Architectural Engineering, Yunnan Communications Vocational and Technical College, Kunming 650500, China

**Keywords:** calcium sulfate whiskers, cement, preparation methods, mechanical properties, durability

## Abstract

In recent years, significant attention has been paid to the use of calcium sulfate whiskers (CSWs) to enhance the performance of cement-based materials (CBM). This technology has attracted widespread interest from researchers because it enhances the performance and sustainability of CBM by modifying the crystal structure of calcium sulfate. This article summarizes the fundamental properties and preparation methods of calcium sulfate whisker materials as well as their applications in cement, potential advantages and disadvantages, and practical applications and prospects. The introduction of CSWs has been demonstrated to enhance the strength, durability, and crack resistance of CBM while also addressing concerns related to permeability and shrinkage. The application of this technology is expected to improve the quality and lifespan of buildings, reduce maintenance costs, and positively impact the environment. The use of CSWs in CBM represents a promising material innovation that offers lasting and sustainable advancement in the construction industry.

## 1. Introduction

Ordinary Portland cement stands as one of the most crucial materials in the construction industry. However, its production and usage have substantial environmental impacts, coupled with drawbacks such as low tensile strength, limited tensile strain, and susceptibility to cracking. To enhance the sustainability of cement-based materials (CBM), researchers have actively explored various modification techniques to improve cement performance. The use of fiber materials, including steel, carbon, glass, and polypropylene fibers, has been explored to enhance the performance of CBM [1]. However, these fiber materials are not without their drawbacks. The fibers are relatively thick in volume, which can lead to the formation of large voids and present challenges in achieving effective interfacial bonding with CBM. Additionally, steel fibers are prone to agglomeration during construction [2]. Consequently, the application of certain microfibers has gathered increasing attention from researchers and has yielded promising results. Nevertheless, fibers such as carbon fibers, renowned for their high tensile strength and elastic modulus, are expensive and exhibit poor dispersion. Glass fibers are susceptible to silicate erosion and may be affected by highly alkaline cement hydration products [2,3].

Some researchers have observed an inverse relationship between the strength of fibers and their diameter. In other words, micro/nanofiber materials with high strength, high elastic modulus, and a large aspect ratio can effectively perform micro-reinforcement tasks [4]. Calcium sulfate whiskers (CSWs) have high aspect ratios. Compared to granular and short-fiber fillers, they demonstrate advantages such as stable particle size, high strength, good toughness, high-temperature resistance, and resistance to chemical corrosion. Currently, several methods are available for preparing CSWs, including ion exchange, microemulsion, ultrasonic, hydrothermal, atmospheric acidification, and microwave. Among these, hydrothermal and atmospheric acidification have been extensively investigated. The primary raw material used for CSWs preparation is gypsum [5]. China currently has the world’s largest proven gypsum reserve, estimated to be 100 billion tons. Furthermore, research suggests that industrial byproducts such as phosphogypsum, calcium salts, sulfur salts, converter slag, tin tailings, and rare-earth tailings can be utilized in the preparation of CSWs. This approach contributes to environmental protection and fosters sustainable development [6]. Therefore, CSWs are considered a nontoxic, environmentally friendly, and cost-effective inorganic material with excellent reinforcing and toughening capabilities [7].

Wan et al. [8] confirmed the role of CSWs in refining the pore structures of cement mortar. The results indicate that the addition of 5 wt.% to CSWs increased the flexural and compressive strengths of the mortar specimens by 28.3% and 8.5%, respectively. It was also observed that the CSWs effectively delayed the formation of microcracks and restricted their propagation. Li et al. [9] found that the addition of 0–1.0% (mass fraction) of CSWs had little impact on the fluidity, initial setting time, and final setting time of cement. Additionally, compared to calcium carbonate whiskers, CSWs had a more significant effect on the strength of Portland cement. Cao et al. [10] investigated the impact of CSWs on the properties and microstructure of cement-based composite materials and compared them with nano-silica. The results showed that the optimum effect was achieved with a 1% addition of CSWs, leading to increases in flexural strength, split tensile strength, and fracture toughness of 79.7%, 34.8%, and 28.7%, respectively. Moreover, these properties, as well as shrinkage deformation and the capillary water absorption coefficient, were superior to those achieved with nano-silica. Zhang et al. [11] studied the combined effect of CSWs and basalt fibers on chloride ion migration in concrete. The results indicated that the addition of 3.0 kg/m^3^ of CSWs combined with 4.5 kg/m^3^ basalt fibers resulted in a more significant improvement in concrete strength and resistance to chloride ion penetration. Cheng et al. [12] investigated the influence of CSWs addition on the high-temperature performance of calcium aluminate cement. When 0–5% of CSWs was added to the cement paste, the performance of the cement paste remained stable. Moreover, with a 4% addition of CSWs, the compressive strength of samples cured at 50 °C and then subjected to high-temperature (500 °C) treatment increased by 25.93% on the 14th day, and the tensile strength increased by 93.63%. In addition, CSWs are widely used as reinforcing agents in rubber, plastics, adhesives, friction materials, papermaking, and for environmental protection. Owing to its relevance beyond CBM, further elaboration of these applications is not provided here [13].

In summary, as an additive for CBM, CSWs have garnered widespread research interest and found industrial applications, presenting revolutionary prospects [14]. The introduction of CSWs effectively enhances the strength, durability, and crack resistance of CBM. This not only improves the quality of construction structures and extends their lifespan, but also plays a positive role in environmental protection by reducing carbon emissions associated with cement production [15]. However, most researchers have predominantly focused on research-oriented discussions regarding the preparation and application of CSWs without providing comprehensive and integrated summaries of various aspects.

This paper discusses the properties and preparation methods of CSWs, their application and mechanisms in CBM, the advantages and disadvantages of their use in CBM, and their practical applications and prospects. Through this review, we examine the applicability of CSWs, optimal addition amounts, and potential technical challenges, providing readers with a comprehensive understanding of this intriguing field. This will facilitate a better understanding of the application of CSWs in CBM, contribute to the advancement of the construction industry, and offer robust, durable, and sustainable solutions for future construction projects.

## 2. Preparation Methods and Basic Properties of Calcium Sulfate Whisker Materials

### 2.1. Preparation Methods of CSWs

CSWs preparation involves the conversion of granular gypsum (calcium sulfate dihydrate) into fibrous, anhydrous calcium sulfate. Equations (1)–(4) represent the process of solution dissolution and dissociation equilibrium [16]. In the subcritical system, SO_3_^2−^ undergoes oxidation by O_2_ in the suspension to form SO_4_^2−^ (Equation (5)). As the concentrations of Ca^2+^ and SO_4_^2−^ increase to reach saturation, CSWs spontaneously nucleates and crystallizes (Equation (6)). The chemical reaction used for synthesizing CSWs is as follows [17]:

CaSO_3_(S) → Ca^2+^ + SO_3_^2^ (1)

H_2_O → H^+^ + OH^X^ (2)

SO_3_^2−^ + H^+^ → HSO^3−^ (3)

2HSO^3−^ + 2H^+^→H_2_SO_3_ → SO_2_↑ + H_2_O (4)

2SO_3_^2−^ + O_2_ → 2SO_4_^2^ (5)

Ca^2+^ + SO_4_^2−^ + nH_2_O → CaSO_4_·nH_2_O (6)

Whiskers are a special type of single crystal, and their growth exhibits distinct characteristics similar to those of crystals. Whisker growth morphology is primarily influenced by factors such as the relative growth rates of different crystal faces, surface energy, internal crystal structure, and environmental conditions. In a freely growing system, variations in the nucleation and growth rates occur because of differences in the various crystal faces. After the growth of stable nuclei, the surface of each crystal nucleus exhibits anisotropy. The methods for preparing CSWs include hydrothermal, atmospheric acidification, ion exchange, microemulsion, and microwave methods [18]. Next, the different preparation methods for CSWs are described.

#### 2.1.1. Preparation of CSWs by the Hydrothermal Method

The main process of the hydrothermal method is as follows: First, in a pressure vessel, the gypsum suspension is transformed into fine, needle-like hemihydrate gypsum under saturated vapor pressure. CSWs are obtained after crystal stabilization treatment [19,20].

The hydrothermal method involves transforming raw materials with a high calcium content into calcium sulfate dihydrate and preparing them in a certain concentration of suspension. This suspension is then placed into a high-pressure reaction vessel, where, under certain pressure and temperature conditions, calcium sulfate dihydrate transforms into needle-like hemihydrate calcium sulfate crystals. Finally, anhydrous CSWs are obtained by high-temperature drying [21]. The advantages of this method are its high whisker conversion rate and relatively simple processing.

**Figure 1:**
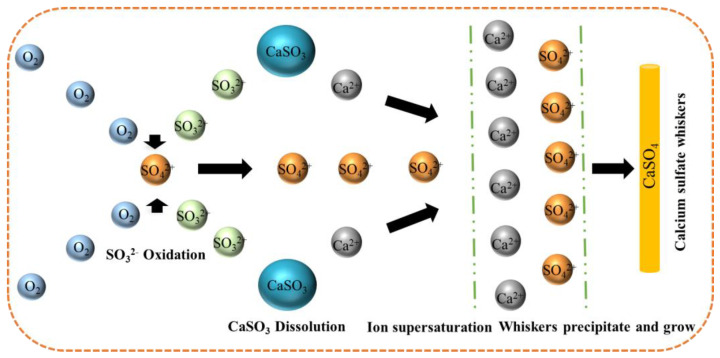
One-step oxidation of CaSO_3_ to prepare CSWs [28].

In the hydrothermal method, water plays multiple roles, serving as a solvent, whisker growth promoter, and medium for pressure transmission [22]. However, the hydrothermal method faces some challenges, with primary issues including lower dissolution and crystallization rates. This necessitates the use of high-pressure reaction vessels to accelerate the infiltration reaction and control the physicochemical factors for the formation and modification of CSWs. Liu et al. [23] used desulfurization gypsum as the raw material to study the effects of desulfurization gypsum particle size, slurry concentration and additives on the desulfurization effect. The effects of three different additives, magnesium chloride, citric acid, and sodium dodecyl benzene sulfonate, on the synthesis of CSWs were explored. The results show that magnesium chloride will reduce the average aspect ratio and aspect ratio of CSWs, and a small amount of citric acid or sodium dodecyl benzene sulfonate can improve the morphology of CSWs. Moreover, when the citric acid concentration is 0.3% and the SDBS concentration is 0.2%, the average length of CSWs reaches 71 μm, and the aspect ratio reaches 66, both of which are 20% higher than the CSWs prepared in the additive-free test. Yang et al. [24] used purified flue gas desulfurization gypsum as raw material to explore the influence of crystal modifier dosage, reaction temperature, pH and other factors on the CSWs crystal morphology. The results show that the dosage of crystal modifier (K_2_SO_4_) and reaction temperature have a significant impact on the morphology and aspect ratio of CSWs, while pH and reaction time have less impact. When the dosage of K_2_SO_4_ is 3 wt.% and the pH is 2, CSWs with excellent quality can be produced by reacting at 130 °C for 60 min. This process increases the production costs. Another limiting factor is the typically low raw-material concentration, which leads to longer reaction cycles. This restricts the industrial application of the hydrothermal method. The specific steps for synthesizing CSWs using the hydrothermal method include the introduction of a gypsum suspension with a mass fraction of approximately 4–6 wt.% into a high-pressure reaction vessel. Under saturated vapor pressure, calcium sulfate particles gradually transform into fibrous hemihydrate gypsum with the assistance of crystal additives. Finally, through evaporative water loss, it transforms into crystal-stabilized CSWs [25]. This process increases the production costs. Another limiting factor is the typically low raw-material concentration, which leads to longer reaction cycles. This restricts the industrial application of the hydrothermal method. Under hydrothermal conditions, crystal growth occurs freely but is influenced by various factors, including reaction temperature, heating rate, pressure, and the pH value, type, and concentration of additives, among others. These factors collectively affect the nucleation and growth of CSWs, as well as the morphology of different crystal faces, necessitating fine adjustments in practical operations to meet specific requirements [17]. Therefore, although the hydrothermal method has the advantage of providing a unique approach for preparing CSWs, technical and economic challenges must be overcome. Luo et al. [26] studied the synthesis of CSWs using sodium sulfate and calcium chloride as raw materials. In their experiment, a 0.6-mol/L calcium chloride solution was slowly added dropwise into a sodium sulfate solution. The molar ratio of Na_2_SO_4_ to CaCl_2_ was 1:8, and the pH was maintained at 6.12. Under these conditions, they successfully obtained CSWs with an aspect ratio of 97.5 and a well-defined morphology. Wang et al. [27] used calcium carbide slag as a raw material and employed the hydrothermal method to prepare different types of CSWs. They investigated the impact of preparation parameters on the formation of CSWs and found that the optimal conditions for preparation were a suspension concentration of 4%, hydrothermal reaction time of 10 h, reaction temperature of 130 °C, and calcium–magnesium ratio of 12:1. Interestingly, at a very low solution pH, nanoscale CSWs with an aspect ratio of approximately 60–80 were observed. As depicted in Figure 1, the flow chart for preparing CSWs through hydrothermal synthesis involves passing O_2_ into a suspension of SO_3_^2−^ in CaSO_3_ to oxidize CaSO_3_ into SO_4_^2−^. Simultaneously, the concentration of Ca^2+^ is increased, inducing the spontaneous crystallization of CSWs.

#### 2.1.2. Preparation of CSWs by Normal Pressure Acidification Method

The atmospheric acidification method is another process for preparing CSWs. The main procedure involves adding an acidic solvent to gypsum raw materials; CSWs are obtained by controlling parameters such as temperature, reaction time, and pH [29].

In atmospheric acidification, the typical practice involves reacting natural gypsum, lime, or slaked lime with sulfuric acid or waste acid to synthesize calcium sulfate dihydrate. Under specific temperatures and acidic conditions, calcium sulfate dihydrate transforms into needle-shaped or fibrous hemihydrate CSWs. Finally, anhydrous CSWs are obtained by high-temperature drying. 

**Figure 2:**
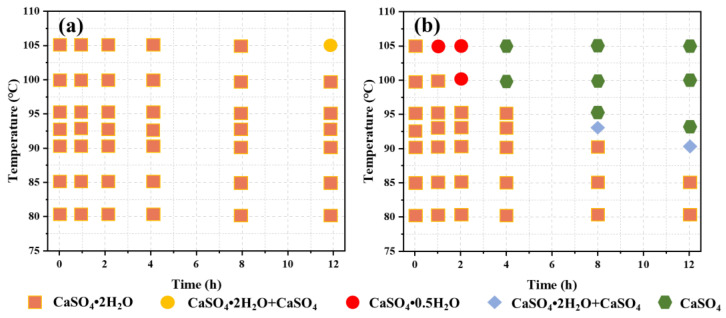
The phase change of CaSO_4_·2H_2_O at different temperatures and duration. (**a**) Hydrochloric acid concentration: 1 mol·L^−1^; (**b**) Hydrochloric acid concentration: 2.5 mol·L^−1^ adapted from [18].

Compared to the hydrothermal method, the atmospheric acidification method has some advantages due to its mild reaction conditions (atmospheric pressure and temperatures below 90 °C) [30,31]. The atmospheric acidification method does not require the use of a pressurized reactor, and the high concentration of calcium sulfate in the raw material significantly reduces production costs. However, despite the advantages of simplicity in recycling waste materials and straightforward manufacturing requirements, atmospheric acidification has limiting factors that restrict its application in large-scale CSW production. Possible limiting factors include the following: Typically, the solubility of raw gypsum in water is extremely low, and its solubility increases significantly in acidic media. This is not conducive to the formation of supersaturated solutions, affecting crystal nucleation and growth, leading to a decrease in both product yield and quality. The atmospheric acidification method for preparing CSWs is carried out in a strongly acidic environment, which makes the acidity of the solution challenging to control. Additionally, the high acidity of the mother liquor is corrosive to equipment and pipelines, which is not conducive to subsequent operations [32]. The atmospheric acidification method for preparing CSWs requires hot filtration, in which crystals grow in a hot solution. The reaction temperature is difficult to control, with a significant heat loss during the reaction. Compared with the hydrothermal method, the growth and aging times of CSWs are longer in the atmospheric acidification method [33]. Sun et al. [33] synthesized CSWs using the atmospheric acidification method and investigated the influence of hydrochloric acid concentration and leaching temperature on the morphology of CSWs. The results showed that when the HCl concentration was 3.7 mol/L and the leaching temperature was 70 °C, CSWs with high purity and a high length-to-diameter ratio could be prepared. Ma et al. [34] investigated a method for preparing CSWs using a concentrated calcium nitrate solution. Experimental results showed that dripping a calcium sulfate solution into a concentrated calcium nitrate solution at room temperature could directly synthesize CSWs with a high length-to-diameter ratio of 93.5. The study also compared the effects of microwave heat treatment at 140 °C and 180 °C on the morphology of CSWs. Lei et al. [18] studied a method for preparing CSWs using hydrochloric acid as a leaching solution to modify low-grade manganese ore. This study explored the effects of pH and reaction time on the leaching of manganese ore, as well as the influence of reaction temperature, stirring speed, precipitation time, calcium ion concentration, and sulfuric acid concentration on the morphology of CSWs. Figure 2 depicts a flow chart for preparing CSWs using the atmospheric pressure acidification method. The results indicate that at lower concentrations of hydrochloric acid, the crystallization of CaSO_4_·2H_2_O remains unaffected by variations in temperature and time. However, as the concentration of hydrochloric acid increases, the crystallization of CaSO_4_·2H_2_O correspondingly increases. A notable transition occurs at approximately 100 °C and after about six hours.

#### 2.1.3. Ion Exchange Method

The CSWs ion exchange method is a water treatment technique based on the principle of an ion exchange resin. The ion exchange resin serves as a template, and sulfate acts as a precipitant. The CSWs are prepared by simple mechanical stirring and heating in a water bath. This method has advantages such as simple equipment, low energy consumption, no need to wash the obtained product, and the ability to recycle the ion exchange resin template [35]. Ion-exchange resins, as a type of polymer material, possess exchangeable ion characteristics. In this process, calcium sulfate acts as a regenerator and is used cyclically to regenerate the resin, enabling it to regain its cation adsorption capacity. The key lies in selecting an ion exchange resin suitable for the target cations in water, especially those with a high affinity for metal ions such as calcium and magnesium. The operating steps include passing water-containing cations through an ion exchange resin bed containing CSWs. In this process, calcium ions in the CSWs undergo ion exchange with metal ions in the water, adsorbing metal ions onto the resin. Over time, the resin gradually becomes saturated and loses its ability to adsorb metal ions. To restore the adsorption capacity of the resin, a regeneration operation is performed, wherein calcium sulfate is used to wash the resin, release the metal ions adsorbed on the resin, and render the resin reusable. The CSWs ion exchange method is widely used in the field of water treatment, particularly for water softening and removing calcium and magnesium ions from hard water. Its advantages include simplicity, low cost, and stable effectiveness, making it a popular water treatment method [36,37].

#### 2.1.4. Microemulsion Method

The microemulsion method uses surfactants to form emulsions of two immiscible solvents. This method has been employed to prepare nanoparticles through nucleation, coagulation, aggregation, and heat treatment [38]. Its most significant advantage is its ability to achieve precise control over the size of nanoparticles. Moreover, in microemulsion systems used to synthesize nanomaterials, reagents can be recovered for recycling [30,39,40]. However, current research on the preparation of nanoparticles using the microemulsion method mainly focuses on size control, with relatively less emphasis on controlling the monodispersity of the particles [31,41]. Additionally, the yield of nanoparticles prepared using the microemulsion method is relatively low. Currently, this method is mainly in the laboratory research stage. Application of this approach to large-scale production remains a long-term goal.

Several factors play significant roles in the preparation of nanometer-sized particles using the microemulsion method, influencing the crystal morphology, particle size, and particle size distribution. Some important factors are as follows: (1) The ratio of water to surfactant is one of the key factors influencing the properties of microemulsions. This ratio impacts the stability of the microemulsion and the properties of the formed nanoparticles. (2) Different types of surfactants possess varying properties, such as hydrophilicity and lipophilicity. Their selection and concentration directly influence the formation of microemulsions and the properties of nanoparticles [42]. (3) The addition of other chemicals, such as catalysts and stabilizers, can regulate the reaction process within the microemulsion, thus affecting the preparation process and the properties of nanoparticles. (4) Temperature serves as a crucial operating parameter in the microemulsion method. Varied temperatures can alter the reaction rate, microemulsion stability, and the final morphology and size of nanoparticles [31,41].

**Figure 3:**
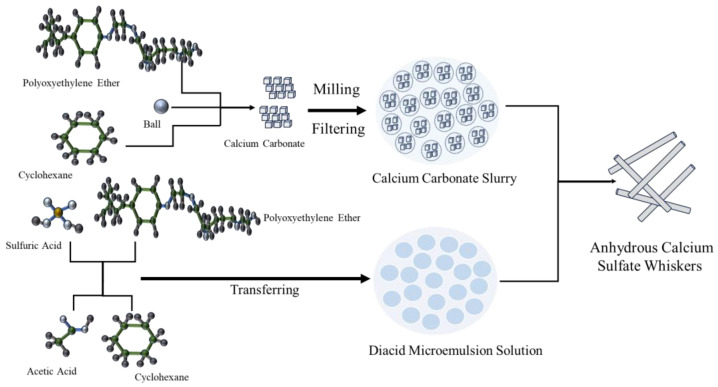
The schematic process of anhydrous calcium sulfate nano whiskers directly synthesized by calcium carbonate and mixed acid through the microemulsion method [39].

Chen et al. [31] utilized a high-concentration calcium acetate solution and dilute sulfuric acid as raw materials and employed a microemulsion solution to prepare anhydrous gypsum calcium sulfate nanowhiskers at room temperature. To further investigate surface modification, organic silicon quaternary ammonium salts and fatty acid methyl ester sulfonates were used to modify calcium sulfate. The surface structure and crystalline evolution of calcium sulfate during the modification process were studied. Figure 3 illustrates a flow chart detailing the preparation of anhydrous CSWs through the direct mixture of calcium carbonate with a solution of mixed acids (glacial acetic acid and dilute sulfuric acid). This method involves creating an acid solution of specific concentration by blending glacial acetic acid and dilute sulfuric acid at room temperature. Subsequently, calcium carbonate is mixed directly with the acid solution to produce calcium sulfate and carbon dioxide. Simultaneously, carbon dioxide escapes into the air, while calcium sulfate crystallizes into CSWs.

#### 2.1.5. Microwave Method

CSWs are prepared using a microwave method using microwave radiation heating to form CSWs using appropriate precursors and conditions. Generally, selecting appropriate precursors is the key to successfully preparing CSWs using the microwave method. This can include compounds containing calcium and sulfate groups, such as calcium sulfate, and other organic or inorganic substances. The selected precursor is dissolved in an appropriate solvent to form a reaction solution. This can include water or other organic solvents, depending on the nature of the precursors and the reaction conditions. The reaction mixture is then heated in a microwave radiator. Microwave radiation can rapidly increase the temperature of a solution, prompting the precursor to react and form CSWs. Under the action of microwave radiation, the precursor undergoes a chemical reaction to generate CSWs. The specific mechanism of the reaction and the product formation pathway may vary depending on the precursor [42]. After preparing the CSWs using the microwave method, the product is separated and purified. The process typically involves filtration, washing, and drying to obtain the final CSWs product. Washing steps can be conducted using various methods, such as repeatedly rinsing with pure water or employing suitable solvents like ethanol or acetone. Drying conditions must ensure thorough drying of the product. Common drying techniques include vacuum drying or heat drying. Temperature and duration should be adjusted according to the specific circumstances to prevent product degradation or loss. Microwave irradiation is an efficient heating method. Compared with conventional heating methods, microwave radiation is an integral heating method that has the advantages of rapid heating, small heat loss, low energy consumption, cleanliness, and no pollution. 

Additionally, phosphogypsum, a bulk solid waste, contains a large amount of calcium sulfate, which makes it a rich source of calcium for CSWs synthesis. Furthermore, combining phosphogypsum with microwaves can effectively enhance the crystallization conversion rate of CSWs. Therefore, the use of phosphogypsum to synthesize CSWs is beneficial for optimizing phosphogypsum utilization. Feng et al. [43] utilized phosphogypsum as the raw material and succinic acid and aluminum sulfate as crystallization converters. They mixed it with a 10% mass fraction of a calcium chloride solution at a solid–liquid ratio of 1:20. CSWs were prepared under microwave conditions, and their crystallization conversion rate reached 96%. The study also found that the microstructure of the CSWs could be well regulated by controlling the microwave radiation time and temperature. This is mainly due to the strong electromagnetic waves generated by microwaves, which cause polarized molecules to experience rotational torque in the electric field, leading to the rearrangement and friction of dipoles, thereby generating a thermal effect and accelerating the formation of the CSWs [44].

**Figure 4:**
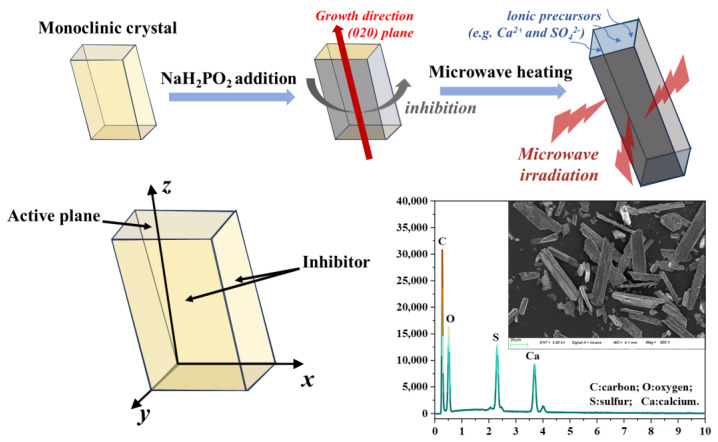
The schematic diagram illustrates the growth of CSWs under microwave radiation, followed by a depiction of the monoclinic lattice of CaSO_4_ seeds. Additionally, an SEM image and energy spectrum of CSWs are presented [45].

Yang et al. [45] successfully synthesized CSWs from wastewater using a microwave-assisted method. The experimental results clearly showed that the introduction of CSWs provided an effective thermal barrier to heat transfer. Compared with the case without adding CSWs, the temperature difference when CSWs are added to the coating exceeds 13 °C, showing excellent thermal insulation performance. The study found that the presence of CSWs can provide a fire barrier in flames up to a temperature of 900 °C. This outstanding performance enhancement is attributed to the endothermic nature of CSWs and their extremely high melting temperature of 1460 °C. Figure 4 depicts a flow chart illustrating the controlled growth of CSWs through microwave radiation. The diagram demonstrates the utilization of microwave heating to expedite the nucleation and crystallization of CaSO_4_, resulting in the formation of numerous monoclinic crystals. Subsequently, NaHPO_2_ serves as an inhibitor, restricting the growth direction of CaSO_4_, thereby facilitating the growth of CSWs in a specific direction and enhancing the aspect ratio.

#### 2.1.6. Comparison of Methods

In general, the most common method for preparing CSWs in the laboratory is the hydrothermal method; however, its industrial application is limited because of its high production cost. Factors affecting the preparation of CSWs by the hydrothermal method include slurry concentration, raw material particle size, initial pH value of the slurry, reaction temperature, reaction time, crystal seeds, crystal form promoters, and crystal stabilizers.

In contrast, the atmospheric-pressure acidification method does not require an autoclave, has a higher mass fraction of raw materials, and significantly reduces production costs. The main factors influencing the preparation of CSWs by this method include the amount of sulfuric acid, reaction time, reaction temperature, type of raw materials, and their ratio with water.

The ion exchange method offers the advantages of simple equipment, low energy consumption, and no need for washing. The ion exchange resin template is then recycled. The main influencing factors for preparing CSWs using this method are the sulfate solution concentration, reaction temperature, reaction time, and stirring rate.

The microemulsion method has the advantages of simple operation and mild conditions. The synthesized product has more advantages in terms of structure and performance than the general physical and chemical methods. It can produce nanoscale CSWs and is, therefore, widely used. The factors influencing the morphology of the CSW synthesized by this method mainly include the reactant concentration, surfactant type, its ratio with water, and aging time.

The microwave method is an efficient and rapid method for preparing CSWs. Whiskers can be formed in a short time using microwave radiation heating. This method has the advantages of simple operation, low energy consumption, and a fast reaction rate and is expected to be widely used in industrial production. The key factors affecting the preparation of CSWs by the microwave method include microwave power, reaction time, reaction temperature, and solution concentration. The microwave method can not only achieve efficient synthesis of CSWs but can also be expected to control the morphology and size of the crystal, allowing it to exhibit superior performance in various application fields. Overall, the microwave method, an emerging CSW preparation method, provides a powerful technical means to improve production efficiency, reduce costs, and optimize product performance.

Table 1 summarizes the advantages, disadvantages, and influencing factors of different CSWs preparation methods. As depicted in the table, the hydrothermal method and the microemulsion method offer higher yields, while the calcium sulfate whisker products obtained from the ion exchange method and the microwave method are of high quality. Furthermore, all preparation methods require consideration of the effects of reaction time and reaction temperature.

#### 2.1.7. Factors That May Affect the CSWs Preparation Process

CSWs are usually grown under specific experimental conditions or from saturated calcium sulfate solutions, and their synthesis is affected by multiple factors. The following factors may affect the calcium sulfate whisker preparation process:

(1) Temperature is a key parameter in whisker growth. Lower temperatures typically facilitate the growth of elongated whiskers, whereas higher temperatures may cause changes in the crystal shape. Controlling the temperature is crucial for adjusting the whisker shape.
(2) The pH of the solution plays a crucial role in the formation and growth of CSWs. Different pH conditions can either promote or inhibit whisker growth.
(3) Solution concentration: CSWs typically grow in supersaturated solutions. Altering the concentration of calcium sulfate in the solution affects the growth rate and whisker morphology.
(4) Ion concentration: The concentrations of other ions dissolved in the solution, such as calcium and sulfate ions, also affect the formation of CSWs. The relative concentrations and ratios of these ions can alter the appearance of whiskers.
(5) Stirring speed: The stirring speed can influence the quality and uniformity of crystal growth during the process. Adequate stirring promotes uniform crystal growth.
(6) Substrate and crystal seeds: Using an appropriate substrate or crystal seed can aid the growth of CSWs.
(7) Time: CSWs growth requires a certain amount of time, and the duration of this process can affect the whisker length and morphology.
(8) Impurities and modifiers: Occasionally, the addition of specific modifiers or impurities can be employed to adjust the morphology of whiskers to better meet specific requirements.
(9) Atmospheric conditions: In certain cases, atmospheric conditions, such as oxygen and carbon dioxide, may influence the formation of CSWs.

### 2.2. Basic Properties and Morphology of CSWs

**Figure 5:**
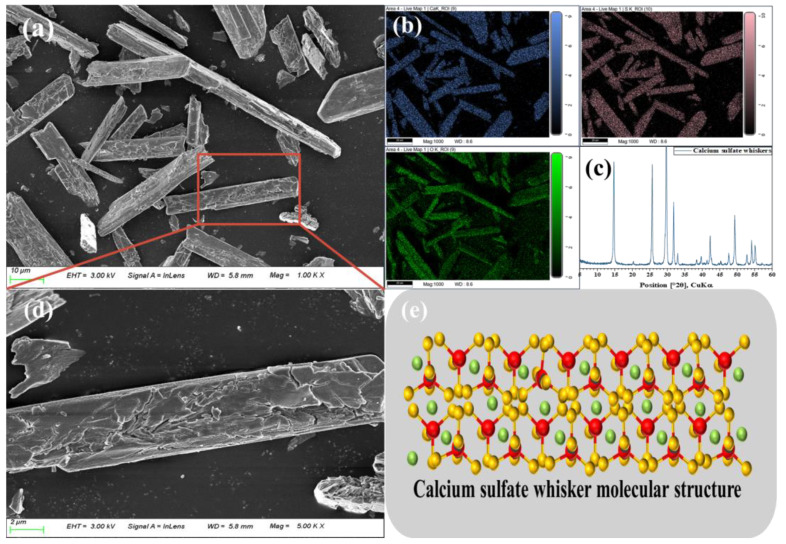
(**a**,**b**,**d**) are the 1000× and 5000× SEM images and energy spectra of CSWs, respectively; (**c**) is the XRD pattern of CSWs; (**e**) is the structural diagram of CSWs.

CSWs (CaSO_4_·× H_2_O) are a distinctive type of calcium sulfate crystals, typically exhibiting a slender, needle-like, or fibrous form, and they usually appear white or transparent. They possess a stable particle size, high strength, good toughness, resistance to high temperatures, chemical corrosion, excellent heat resistance, a high elastic modulus, and favorable processability [50]. The chemical composition of CSWs is similar to that of gypsum and consists of calcium sulfate (CaSO_4_). Their density and melting point are comparable to those of ordinary sulfuric acid. However, unlike typical hydrated calcium sulfate compounds such as gypsum, CSWs predominantly appear in crystalline form. The structure of CSWs includes two water molecules (H_2_O), which exist in the crystal lattice as crystalline water. This sets CSWs apart from common calcium sulfate hydrates, such as gypsum, which usually contain more water molecules in their crystal structures. Furthermore, CSWs themselves have a certain degree of hardness, providing better filler performance than calcium sulfate compounds such as gypsum. However, CSWs are more brittle than other fibers, making them prone to brittle fracture under certain conditions [51]. A comprehensive analysis reveals that CSWs possess the advantages of both fibers and inorganic fillers while sharing the same chemical composition as the gypsum matrix, thus exhibiting excellent compatibility. This unique combination effectively addresses interfacial bonding issues encountered by other fibers in CBM and mitigates common internal defects in fiber-reinforced cementitious composites. The chemical composition and morphology of CSWs are depicted in Figure 5, wherein CSWs predominantly appear as elongated strips primarily composed of calcium, sulfur, and oxygen. The surface of crystallized CSWs exhibits slight irregularities, including a few microcracks and defects, potentially enhancing the interface bonding between CSWs whiskers and CBM [52]. The subsequent section will delve into the differences in mechanical properties between CSWs and calcium sulfate compounds (such as gypsum), ordinary fibers, and provide a discussion on mechanism analysis.

## 3. The Application of CSWs to Cement and the Difference from Gypsum and Fiber

### 3.1. The Role of CSWs in Cement

The utilization of CSWs in cement is a prevalent method for enhancing cement material properties. It is frequently employed to improve the performance of concrete and reduce its dependence on traditional cement. CSWs, which are microfibers derived from calcium sulfate crystals, enhance the mechanical properties, durability, and specific functions of concrete when incorporated into cement [8,53].

**Figure 6:**
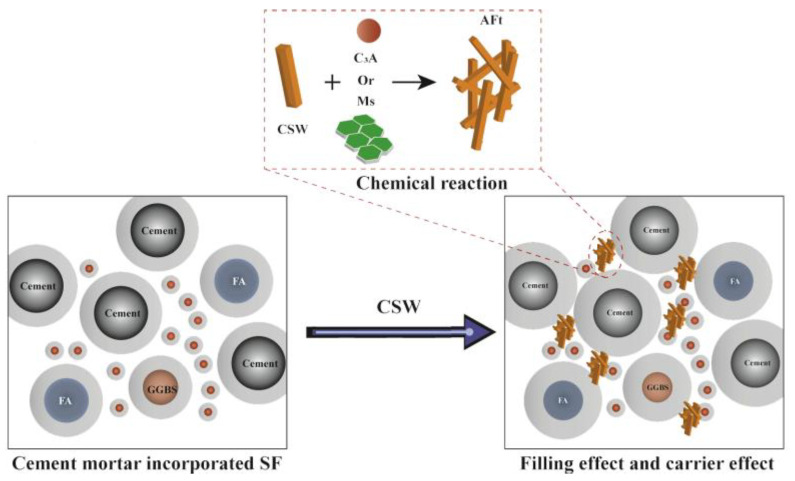
Microscopic mechanism of partial replacement of silica fume (SF) by CSWs: cement mortar mixed with SF, and cement mortar in which CSWs partially replaces SF [55].

CSWs can create a fibrous structure within the cement matrix, resulting in denser and more compact concrete. They effectively absorb and disperse stress within concrete, thereby enhancing its toughness. The presence of CSWs when concrete is under stress mitigates crack expansion, thereby improving the overall toughness and reducing the likelihood of brittle failure [54]. This contributes to an increase in the compressive strength of the concrete, enhancing its durability under stress. Cao et al. [10] demonstrated a 10.3% increase in compressive strength and a 10.2% improvement in the fracture toughness in cement-based composites with the addition of 2% of CSWs. When the CSWs content was 1%, the flexural strength and fracture toughness increased by 10.3% and 10.2%, respectively. The tensile strength and fracture energy increased by 79.7%, 34.8%, and 28.7%, respectively. Wan et al. [8] applied a new type of microfiber material, CSWs to the cement matrix to enhance the strength of cement-based composite materials. CSWs were found to effectively delay the formation of microcracks and limit their expansion. The interaction mechanisms between the CSWs and steel bars were similar and primarily manifested in three aspects: whisker pullout, crack deflection, and crack bridging. Moreover, they could significantly optimize the pore size, increasing harmless pores from 9.33% to 10.62% and reducing harmful pores from 2.08% to 1.90%. The fiber structure formed by CSWs in concrete bridges microscopic cracks, preventing further expansion in the CBM. The bridging effect contributes to preserving the integrity of the concrete and enhancing its tensile and flexural strengths, particularly when subjected to tensile stress. Li et al. [9] compared CSWs with calcium carbonate whiskers, and the results indicated strength increases of 19.54% and 35.84% upon the incorporation of 0.4% and 1.0% CSWs, respectively. Furthermore, CSWs exhibited a greater ability to enhance the strength of ordinary Portland cement compared to calcium carbonate whiskers. The incorporation of CSWs not only retards the aging process of concrete but also extends the service life of the structure. Moreover, the addition of an appropriate amount of CSWs enhances the mechanical properties of concrete by facilitating cement hydration reactions and promoting the formation of additional hydration products. The CSWs may serve as a catalyst to enhance the hydration of cement particles, leading to the formation of a stronger gelling system. However, excessive CSWs addition decelerated the hydration reaction of cement, thereby prolonging the setting time of the concrete. Figure 6 presents the microscopic mechanism diagram following the substitution of silica fume with CSWs. The illustration demonstrates that the incorporation of CSWs yields both physical and chemical benefits. Given the chemical resemblance between CSWs and gypsum, their addition enables dissolution and reaction with C_3_A to generate monosulfates or ettringite. Furthermore, the distinctive fiber characteristics of CSWs serve as nucleation sites for cement hydration, thereby facilitating hydration reactions.

**Figure 7:**
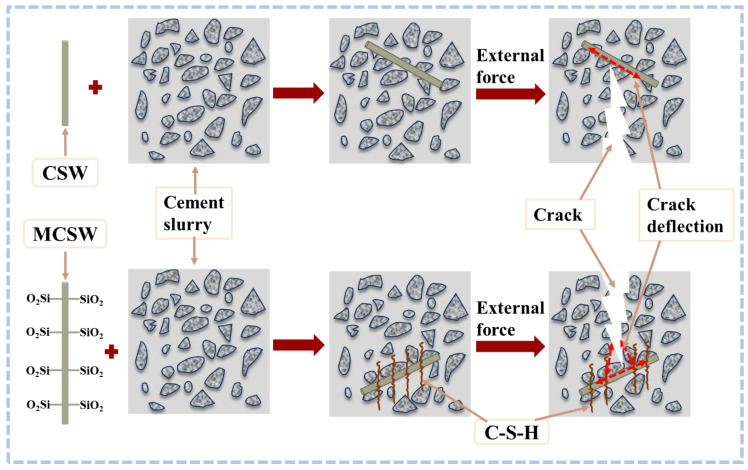
Schematic of the CSWs-reinforced cement stone mechanism [60].

The incorporation of CSWs effectively enhances the crack resistance of concrete and prevents crack expansion. This is attributed to the network structure formed by CSWs in concrete, which effectively withstands cracks induced by temperature changes and shrinkage, thereby reinforcing the overall structural stability of the concrete [56]. Moreover, in the event of microcracks occurring in concrete, CSWs impede further propagation of cracks through their fiber network structure, thereby preserving the overall strength and durability of the concrete. Wu et al. [57] demonstrated that, as a result of the hydration reaction triggered by CSWs, more hydration products were produced, which enhanced the interfacial bonding effect of CSWs compared to basalt fibers. This, in turn, led to a more effective refinement of the pore size and an overall improvement in durability. CSWs enhance the durability of concrete, rendering it resistant to chemical and environmental attacks. This heightened durability is predominantly manifested in the ability of concrete to withstand corrosive substances, such as sulfates, chloride ions, and acids. The incorporation of CSWs aids in controlling the shrinkage of concrete and mitigating the shrinkage resulting from cement hydration. This has a positive impact on enhancing the overall stability of concrete, minimizing internal stress, and reducing the occurrence of cracks. CSWs can be employed to formulate high-performance concrete with superior engineering properties [58]. This type of concrete offers more pronounced advantages for projects that require high structural performance, such as bridges, tunnels, and other buildings. The incorporation of CSWs into cement plays a crucial role in controlling temperature-induced cracks in concrete. During high-temperature seasons or large-volume concrete pours, CSWs can attenuate the temperature changes in concrete, effectively mitigating the occurrence of temperature-induced cracks and enhancing the overall stability of the structure [59]. The incorporation of CSWs has a substantial impact on the freeze–thaw resistance of concrete. CSWs improve the microstructure of concrete and reduce water penetration, thereby minimizing damage during freeze–thaw cycles and enhancing durability, particularly in cold regions. Zhang et al. [60] employed ethyl orthosilicate to modify CSWs and harnessed its retarding properties for incorporation into oil-well cement to achieve favorable mechanical properties. Figure 7 illustrates the comparison of surface modification techniques using ethyl orthosilicate to enhance the distribution of CSWs and improve the interface bonding with CBM, subsequently enhancing the mechanical properties of the CBM. The findings indicate that after modification with ethyl orthosilicate, the surface of CSWs exhibits increased generation of C-S-H chains, thereby enhancing the interfacial adhesion between CSWs and the cement matrix.

Owing to their diverse properties, CSWs can find broad applications in various scenarios, including extending the concrete pumping time, producing crack-resistant concrete, formulating high-performance concrete, reducing cement consumption, and minimizing environmental pollutant emissions. In summary, the incorporation of CSWs in cement not only enhances the mechanical properties, durability, and crack resistance of concrete but also addresses the demand for high-performance concrete, providing a more reliable and stable foundation for building structures. However, in practical applications, scientific and judicious proportioning based on the specific engineering requirements and concrete properties must be performed.

### 3.2. Hydration Mechanism of CSWs

The hydration mechanism of CSWs in cement is a complex and crucial process that directly affects their physical and engineering properties. Hydration is a process in which the primary ingredients in cement react with water to form silicate gels and other hydration products that are responsible for the hardening and strength development of concrete [61,62]. As a control agent and additive, CSWs play a crucial role in hydration [63]. Research indicates that sulfate ions have a dual effect on cement hydration, both promoting and retarding it. The presence of a substantial amount of sulfate ions in the solution increases the solubility of calcium silicate and delays the hydration of aluminate [64]. However, it also accelerates the hydration of silicate [62,65,66]. The hydration mechanism of CSWs in cement involves several key processes.

Dissolution of CSWs:

When CSWs (CaSO_4_·× H_2_O) are added to the cement slurry, they initially dissolve, releasing calcium ions, sulfate ions, and H_2_O [67]. This reaction can be expressed as:

CaSO_4_·2H_2_O + 2H_2_O → Ca^2+^ + SO_4_^2−^ + 2H_2_O

Hydration of CSWs:

The dissolved CSWs actively participate in the cement hydration process. The sulfate ions (SO_4_^2−^) in the CSWs react with tricalcium aluminate (C_3_A) in cement to form ettringite, which is a crucial step in concrete hydration [68]. The formation of ettringite refines the pore size, leading to improved strength. However, this may also hinder early cement hydration, thereby delaying the setting of cement to a certain extent, particularly when higher amounts of CSWs are added [69,70]. The chemical formula for this reaction is as follows:

3CaO·Al_2_O_3_ + 3(CaSO_4_·2H_2_O) + 26H_2_O → Ca_6_(Al(OH)_6_)_2_·(SO_4_)_2_·26H_2_O

Post-hydration of CSWs:

In cement concrete, the hydration process of the CSWs does not stop during ettringite production. Over time, ettringite will rehydrate to form monosulfoaluminate [64,71]. This hydration reaction enhances the strength and durability of concrete [72,73]. The chemical formula for this reaction is as follows:

[Al(OH)_4_]^−^ + SO_4_^2−^ + 4Ca^2+^ + aq. → C_4_ASH_18_

Effect of CSWs on cement hydration:

The addition of CSWs influences the cement hydration process. Initially, the presence of CSWs delays the setting time of the cement, particularly when a large amount of CSWs is added [74]. This is advantageous for projects that require longer setting times, such as concrete pumping. Additionally, the hydration process of CSWs enhances the crack resistance of concrete, diminishes shrinkage stress, and lowers the risk of cracking. Finally, the incorporation of CSWs enhances the durability of concrete, reduces its permeability, and consequently extends the life of the structure.

However, the addition of excess CSWs can lead to reduced durability [75]. Specifically, during the later stages, the undissolved CSWs will further dissolve, resulting in the complex chemical and physical problem of sulfate corrosion, which ultimately leads to the deterioration and damage of building components [76,77,78]. The main phenomena that may occur include: (1) Further dissolution of CSWs reducing the pH value in concrete and causing corrosion of steel bars [79,80]; (2) The dissolved sulfate undergoing a secondary hydration reaction with C_3_A, among other compounds, resulting in the production of more ettringite, leading to expansion and cracking of the structure [73,81,82]. Therefore, while CSWs have a beneficial effect on the hydration of CBM, the amount added needs to be optimized and strictly controlled to prevent poor stability and reduced durability in later stages.

**Figure 8:**
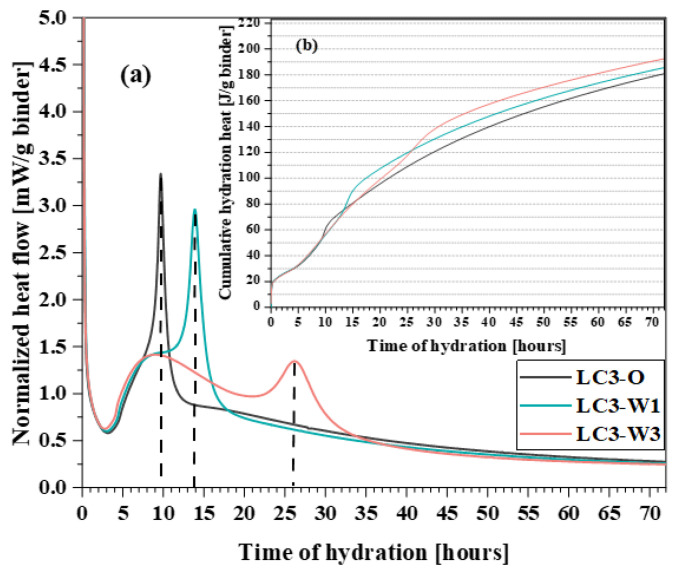
Isothermal calorimetry data: (**a**) heat flow and (**b**) cumulative heat normalized to binder weight.

The heat of hydration experiment showed that the addition of varying amounts of CSWs has varying effects on the hydration process of calcined limestone clay. As shown in Figure 8, the initial hydration exothermic peak is primarily attributed to ion dissolution [71,83]. After the induction period, two distinct exothermic peaks were evident during the acceleration period, corresponding to the hydration reactions of C_3_S and C_3_A. The former primarily produces C-S-H, whereas the latter mainly forms ettringite and monosulfide salts [70,84]. As the amount of added CSWs increased, the C_3_A exothermic peak was delayed, occurring at 10, 14, and 26 h, respectively. This delay is mainly attributed to the increase in the concentration of sulfate ions dissolved by the CSW [85,86]. Additionally, when the CSWs content reached 3%, the cumulative heat of hydration was the highest. This is primarily attributed to the increased presence of calcium sulfate ions, which promote the hydration of the C_3_A component and the formation of greater amounts of ettringite and monosulfate [87,88]. The hydration heat results indicate that the judicious addition of CSWs contributed to the complete hydration of C_3_A, thereby enhancing the performance of the material.

### 3.3. Effect of CSWs on Mechanical Properties

**Figure 9:**
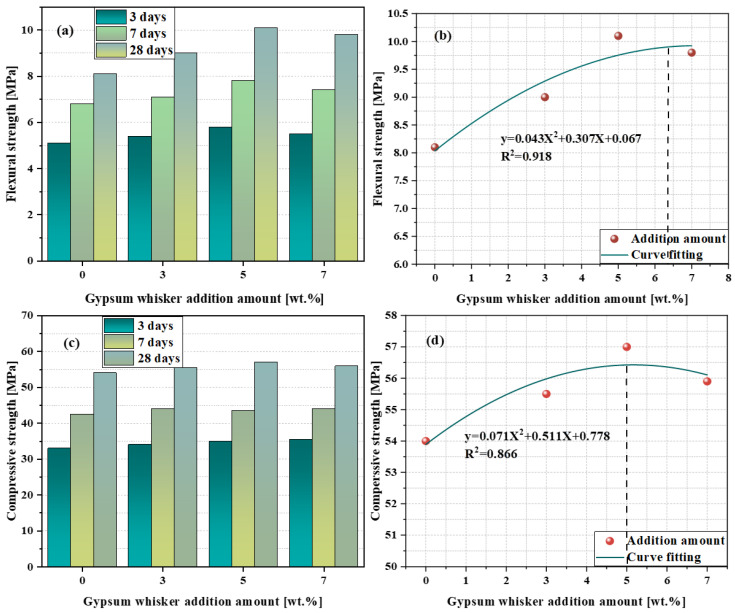
(**a**,**c**) represent the splitting and compressive strengths, respectively, and (**b**,**d**) represent the fitting curves of the splitting and compressive strength, respectively [8].

Figure 9 shows the splitting and compressive strengths with different amounts of added CSWs. Table 2 displays the percentage increase in strength for various CSWs amounts compared with ordinary Portland cement. The data were obtained from the literature [8]. As shown in Figure 9, the addition of 0–7 wt.% of CSWs significantly enhanced both the splitting and compressive strengths. Notably, the improvement in splitting strength was particularly pronounced. After 28 d of curing, the inclusion of 5% CaSO_4_ resulted in a 28.3% increase in strength compared to the control group. This enhancement may be attributed to the formation of a fibrous network structure in the CSWs cement that bridges various components. Furthermore, the dissolution of CSWs can increase the sulfate content and react with C_3_A to generate greater amounts of ettringite and monosulfide salts [89,90]. When the addition amount was increased to 7%, both the flexural and compressive strengths of the sample decreased compared to those of the 5% sample. This may be because (1) as the amount of CSWs increased, the overall consistency improved, and the interface defects between the whiskers and cement paste tended to agglomerate or aggregate; (2) the amount of dissolved sulfate increased, and an excess of sulfate inhibited C_3_S hydration. The results indicated that when the amount of added CSWs was 5%, the mechanical effect was maximized.

### 3.4. Changes in Mechanical Properties of CSWs at High Temperatures

**Figure 10:**
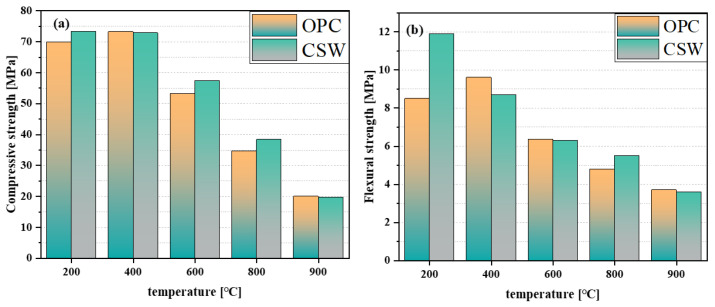
(**a**,**b**) represent the high-temperature splitting strength and compressive strength of undoped CSWs and doped with 2 wt.% of CSW, respectively [10].

Figure 10 depicts the compressive and splitting strengths at different temperatures using data sourced from [10]. Figure 10a,b show that the mechanical properties improved at 200 and 400 °C. This improvement may be attributed to the secondary hydration of unhydrated cement particles in a high-temperature steam environment. At temperatures of 400 and 600 °C, the mechanical properties deteriorated, primarily due to the decomposition of hydration products. Since the decomposition temperature of calcium sulfate at high temperatures generally ranges between 1300 °C and 1600 °C, the strength change at elevated temperatures is mainly controlled by the hydration products generated [91]. In addition, between the temperatures of 200 and 400 °C, the mechanical properties of the sample with added CSWs were weaker than those of the control sample. This may be due to: (1) a dehydration reaction occurring in the CSWs (CaSO_4_·× H_2_O); (2) the partially dissolved sulfate in the CSWs reacting with C_3_A to generate greater amounts of ettringite and monosulfide salts, which undergo decomposition between 200 and 300 °C.

### 3.5. Comparison of CSWs with Gypsum and Fiber

**Figure 11:**
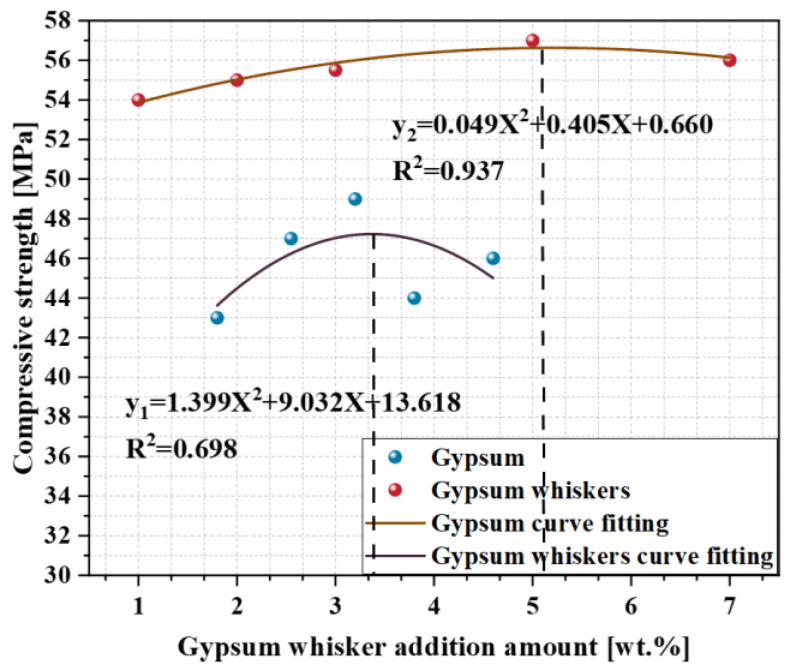
Comparison between the compressive strength of sulfate-added concrete and CSWs-added concrete after 28 days of curing [8,71].

C_3_S and C_3_A are crucial phases in cement, with C_3_A undergoing rapid early hydration reactions. In instances where no gypsum is added or only a minimal amount is included, C_3_A swiftly reacts with water to produce hexagonal sheets of calcium aluminate hydrate, resulting in instantaneous bonding and leading to “Flash setting”. “Flash setting” refers to rapid hardening within a short timeframe, causing a loss of workability. Importantly, the strength of the formed components tends to be low. To prevent flash condensation, it is essential to add an appropriate amount of sulfate [71]. Studies have shown that the dissolved CSWs can also serve as a calcium source for the matrix and influence the setting time of cement. In addition, they hydrate with C_3_A to form ettringite and monosulfide salts, thereby contributing to an overall improvement in strength. From a hydration perspective, the CSWs have an effect similar to that of gypsum. In addition, the incompletely dissolved CSWs provide more nucleation sites and promote hydration reactions. In terms of physical effects, the incompletely dissolved CSWs can fill detrimental pores. The aspect ratio of the CSWs can create a staggered network structure in the CBM, enhancing its mechanical properties. Furthermore, the whisker pulling out, crack deflection, and crack bridging effects of CSWs can effectively delay the formation of microcracks and limit the expansion of such cracks. As shown in Figure 11, after 28 days of curing, the compressive strength of the CSWs sample was significantly higher than that of the gypsum sample when the amount of gypsum added was the same as that of the CSWs.

**Figure 12:**
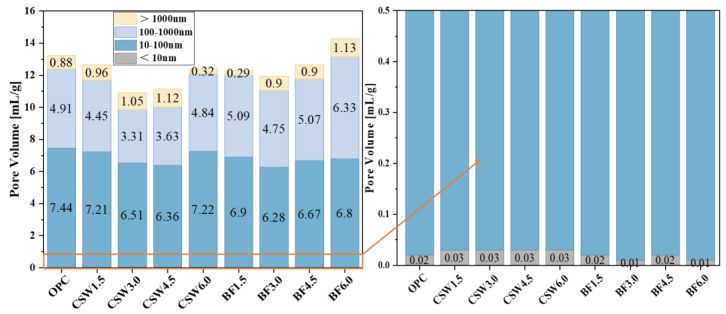
Variation in pore diameter for different types of concrete [11].

As shown in Figure 12, these are the NMR T2 spectra of CSWs concrete and BF concrete, with data sourced from [11]. BF denotes basalt fiber. The addition of an appropriate amount of CSWs exhibited a significantly superior pore size refinement effect compared to that of basalt fiber addition. This may be because (1) the volume of the CSWs was smaller than that of the basalt fibers. At the same volume, the number of CSWs is greater than that of basalt fibers. (2) The dissolution of an appropriate amount of CSWs can increase the sulfate content, generate more ettringite and monosulfide salts, and refine the pore size.

In summary, CSWs not only inherit some of the advantages of gypsum but also exhibit a fiber-enhancing effect. On the one hand, the dissolution of CSWs can provide Ca^2+^ and SO_4_^2−^ ions for the hydration reaction, which react with C_3_A to form ettringite, filling gaps and improving the strength of the specimen. On the other hand, the high aspect ratio and specific surface area of CSWs can effectively reduce the expansion of cracks and the generation of microcracks [92]. Therefore, the addition of an appropriate amount of CSWs can effectively achieve a reinforcing effect. However, the CSWs will not be completely dissolved. The CSWs, which are completely wrapped by the hydration products, may continue to dissolve in the later stage. The continued dissolution of the CSWs will cause the pH value in the concrete to decrease and increase the risk of reinforcement corrosion. At the same time, as the Ca^2+^ and SO_4_^2−^ ions continue to increase, they may react with C_3_A to generate more ettringite in the later stage, which may increase the risk of expansion and cracking of concrete. Additionally, compared with fibers, CSWs themselves have poor dispersion, and their high specific surface area may also reduce the fluidity of concrete and increase the difficulty of construction. Therefore, further study is needed to optimize and enhance the durability of CSWs.

## 4. Potential Benefits of CSWs

The development and application of CSWs as an inorganic whisker material are currently in a vibrant and active phase. Their outstanding physicochemical properties and relatively low manufacturing costs have garnered widespread attention from the industrial sector. CSWs are fibrous monocrystals of anhydrous calcium sulfate with a refined structure, perfect morphology, specific cross-section, and stable dimensions. Typically, their average aspect ratio (The ratio of the length of a whisker to its diameter) ranges from 50 to 80 [26]. Compared to other short fibers, CSWs have several advantages, including high-temperature resistance, resistance to chemical corrosion, good toughness, high strength, ease of surface treatment, compatibility with polymers such as rubber and plastic, and low toxicity [93]. In addition, the cost of CSWs is relatively low, usually priced at only US$1−3 per kilogram, providing unparalleled cost-effectiveness in the whisker market. Because of their outstanding performance and cost-effectiveness, CSWs have widespread applications in various industrial production fields. This extensive use has solidified the position of CSWs in the industrial sector, offering reliable solutions for material requirements in various fields [94].

### 4.1. Optimal Utilization in the Construction Industry

Controlling the setting time of cement: CSWs can be employed to retard the setting time of cement. The addition of an appropriate amount of CSWs can extend the setting time of the cement, which is particularly useful for large-scale projects and concrete transportation [67,74].

Enhancing the crack resistance of cement: The application of CSWs improves the crack resistance of cement. This can reduce the shrinkage stress in the cement, thereby lowering the risk of cracking, particularly in concrete structures. This is crucial for maintaining structural stability [95].

Reducing the environmental impact of cement production: CSWs can serve as a substitute for cement clinker, thereby reducing the demand for natural resources. Additionally, they can help decrease sulfur dioxide emissions, making a significant contribution to environmental protection.

Extending the concrete pumping time: In large-scale concrete construction projects, transporting concrete to the construction site by pumping is a common practice. However, prolonged transportation may cause concrete to set, thereby impacting construction progress [56,67,96]. Engineers can incorporate CSWs into concrete to extend the pumping time.

The role of CSWs is to slow the hydration reaction of cement, thereby extending the setting time of the concrete and ensuring smooth pumping and pouring. This application ensures that the concrete maintains its fluidity during transportation and pouring, thereby reducing the problems and delays in the construction process.

Cracking is a common problem in concrete structures, particularly in dry and high-temperature environments. Engineers can use CSWs to enhance the crack resistance of concrete. The presence of CSWs in concrete can reduce shrinkage stress and decrease the risk of cracking. The result of this application is an improvement in the durability of concrete structures, thereby reducing maintenance costs and the need for repair.

One of the approaches for enhancing the properties of high-performance concrete with the aim of improving its durability and resistance to permeability while maintaining its strength is the addition of CSWs. They can be used as a control agent to enhance the properties of high-performance concrete. The addition of CSWs improves the engineering properties of concrete, reduces its permeability, and extends its life. The result of this application is the production of more durable and reliable high-performance concrete that is suitable for long-term and demanding engineering projects [97].

Reducing cement usage: To diminish the dependence on natural resources and decrease carbon emissions, some engineering projects have adopted strategies to reduce cement usage. CSWs can be used as a replacement material to reduce cement requirements. This not only reduces project costs but also benefits the environment. The results of this application are a reduction in cement usage, a lower carbon footprint, and the promotion of sustainable construction.

Reducing the emission of environmental pollutants: In the flue gas desulfurization process of some coal-fired power plants, waste containing CSWs is produced [98]. By recycling these wastes and incorporating them into cement, not only can waste treatment costs be reduced but sulfur dioxide emissions can also be minimized, contributing to environmental protection. The result of this application is a reduction in air pollutant emissions, which contributes to improved environmental quality.

### 4.2. In the Field of Environmental Protection

Waste recycling: The application of CSWs promotes waste recycling and reuse. In particular, in the treatment of coal mine waste and flue gas desulfurization in coal-fired power plants, the generated calcium sulfate waste can reduce the cost of waste treatment and minimize environmental pollution [99].

Reducing carbon dioxide gas emissions: The application of CSWs can also mitigate carbon dioxide gas emissions in industrial processes, thereby improving air quality and the environment.

### 4.3. Advantages and Disadvantages in the Field of Building Materials

The application of CSWs contributes to sustainable construction, reducing dependence on limited natural resources, lowering the carbon footprint, and promoting environmentally friendly architecture and construction. Compared with traditional concrete additives, the preparation process of CSWs is generally more environmentally friendly, reducing the negative impact on the environment. Additionally, by improving the properties of concrete, a building’s energy consumption can be reduced, thus lowering carbon emissions. Adding an appropriate amount of CSWs can effectively reduce the internal pore structure of concrete materials and refine the pore size [10]. This reduction minimizes the penetration of moisture, pollutants, and ion erosion, thereby enhancing the indoor environmental quality and living comfort of the building. The addition of CSWs may help to slow down the hardening process of CBM, providing longer construction times, which could be beneficial for certain projects [85,86]. CSWs can also be utilized to enhance the properties of building materials, including improving the strength, durability, and crack resistance of concrete. It can enhance the resistance of CBM to certain chemical corrosion and improve performance in acidic or alkaline environments. Additionally, it aids in the hydration reaction of CBM, leading to the formation of a denser and more uniform structure. Moreover, the addition of an appropriate amount of CSWs to concrete can enhance its durability and strength, thus extending the service life of the building. This helps to reduce the building’s maintenance needs and frequency of renewal, ultimately increasing its sustainability [56,57].

In actual engineering applications, the addition of CSWs may pose challenges to certain engineering implementations, such as concrete mixing and pouring. The inclusion of CSWs could potentially lead to settling issues, necessitating the use of special dispersants to ensure uniform dispersion within CBM. In some instances, the presence of CSWs may result in a decrease in the short-term strength of CBM, necessitating comprehensive consideration of engineering requirements [86,87]. Additionally, the addition of CSWs may introduce complexity to the preparation of CBM and require stricter production controls. The addition of CSWs may indeed affect the wear resistance of CBM, and it is essential to balance various performance requirements in the design. Moreover, adding CSWs can impact the fluidity of cement, potentially making the concrete more challenging to handle during construction. This could adversely affect construction efficiency and the uniformity of the concrete.

Concerning durability and stability issues, the addition of CSWs to cement may indeed lead to expansion problems. This is due to the moisture absorption and expansion of CSWs within the cement matrix, potentially negatively impacting the stability of the structural material. Furthermore, it is correct that the presence of excess CSWs may trigger a post-hydration reaction, exacerbating these concerns. In the later stages, undissolved CSWs may continue to dissolve, leading to the complex chemical and physical issue of sulfate corrosion [100]. This can result in the deterioration and damage of building components. Possible phenomena mainly include: (1) Further dissolution of CSWs will cause corrosion of steel bars; (2) Additional sulfates may react with compounds like C_3_A to produce more ettringite, leading to expansion and cracking of the components [101,102]. Therefore, while CSWs have a beneficial effect on the hydration of CBM, the amount of addition needs to be carefully optimized and strictly controlled to prevent compromised stability and reduced durability in the later stages [103,104,105].

Although CSWs can enhance the sulfate attack resistance of cement, it may have a negative impact on the strength of concrete in some cases. This may depend on the CSWa content and the specific formulation of the concrete [106]. 

## 5. Practical Applications and Prospects

CSWs have numerous advantages, including high strength, high modulus, high toughness, high insulation, wear resistance, high-temperature resistance, acid and alkali resistance, corrosion resistance, good infrared reflectivity, easy surface treatment, easy compounding with polymers, and nontoxicity. Their physical and chemical properties have found wide applications. Currently, the raw materials used for the preparation of CSWs are inexpensive and readily available, for example, industrial waste. However, most processes require heating and pressurization and have high energy consumption. Therefore, exploring and developing new and simpler processes with low energy consumption is a direction for future research. Moreover, the preparation of CSWs with higher aspect ratios should be studied.

### 5.1. Applications in the Construction and Cement Industries

CSWs have been widely utilized in the cement industry as a control, anticracking, and retardant. They can be employed to control cement setting time, enhance cement crack resistance, reduce environmental pollution, and improve concrete properties. The application of CSWs is likely to become widespread, particularly in the field of sustainable construction. They can be used to reduce cement usage and carbon emissions and promote green buildings and construction.

### 5.2. Applications in the Field of Environmental Protection

CSWs have been employed in environmental fields to reduce carbon dioxide emissions from industrial processes, improve air quality, and reduce waste disposal costs. Environmentally friendly applications of CSWs may extend beyond contributing to the reduction of pollutant emissions, improving air quality, and mitigating the impact of industrial waste.

### 5.3. Applications in the Field of Building Materials

CSWs have been widely utilized in construction materials to enhance concrete properties, such as strength, durability, and crack resistance.

The applications of CSWs are likely to expand further, including in the development of new building materials, contributing to the development of more durable and environmentally friendly buildings.

Overall, CSWs have achieved significant success in practical applications and have broad development prospects. As sustainability and environmental protection awareness continue to increase, CSWs will play a greater role in various fields, aiming to reduce resource wastage, minimize environmental impacts, and promote sustainable development.

In addition, despite a certain research basis for the application of CSWs in CBM, there are relatively few studies on it in some emerging fields, such as sustainable construction and biomaterials. This may be attributed to the evolving demands in these areas and the fact that the potential applications of CSWs in these fields have not yet been fully explored.

Furthermore, while the advantages of CSWs in CBM have been widely discussed, their disadvantages and limitations have received comparatively less attention. Research on potential issues that may arise in engineering practice, such as compatibility with other materials and long-term performance stability, still requires in-depth investigation.

Therefore, introducing research on CSWs in emerging fields and conducting an in-depth exploration of their advantages, disadvantages, and potential applications will help expand their application scope in the field of building materials and promote innovative development in related fields.

## 6. Conclusions

The application of CSWs in the CBM is an area of extensive research, with the main purpose of improving the performance and properties of these materials. This article discusses different preparation methods for CSWs, hydration mechanisms, reinforcement mechanisms in the CBM, and prospects for the application of CSWs in the CBM. The relevant summary is as follows:

(1) CSWs are primarily produced by hydrothermal and atmospheric-pressure acidification, ion exchange, microemulsion, and microwave methods. The raw materials required include high-calcium industrial solid waste.
(2) As a reinforcing material, CSWs can significantly enhance the tensile strength, compressive strength, and durability of the CBM, thereby improving their load-bearing capacity.
(3) The judicious use of CSWs can effectively reduce the shrinkage of the CBM, enhance the volume stability of its materials, and mitigate the occurrence of cracks. This improves material durability and reduces structural maintenance costs. The optimal amount for achieving the best improvement in the mechanical properties is typically in the range of 4 to 6 wt.%.
(4) The presence of CSWs affects the microstructure of the CBM, forming a dense lattice structure and enhancing the overall performance of the material.
(5) The use of CSWs helps decrease dependence on natural resources and enhances the environmental friendliness of the CBM.

## Figures and Tables

**Table 1:** Comparison of the advantages, disadvantages and influencing factors of different CSWs preparation methods.

Preparation Method	Advantage	Disadvantages	Influencing Factors	References
Hydrothermal	High yield, good controllability, wide application range, etc.	High energy consumption, high reaction condition requirements, poor product dispersion, etc.	Reaction temperature, reaction time, reactant concentration, additives, reaction pressure, etc.	[5,22,25,28]
Normal pressure acidification method	Low cost, environmentally friendly, wide applicability, etc.	The reaction time is long, the output rate is low, and the product morphology is difficult to control, etc.	Acidifying agent concentration, reaction temperature, reaction time, selection of calcium source and calcium sulfate source, stirring rate, reaction vessel and equipment, etc.	[17,46,47]
Ion exchange method	High product quality, simple operation, wide application range, etc.	Higher cost, longer response time, high equipment requirements, etc.	Ion exchange medium, reaction temperature, reaction time, reactant concentration, stirring rate, pH value, etc.	[35,36,48]
microemulsion method	Simple operation, high yield, good product dispersion, etc.	The preparation conditions are high, the cost is high, and it is affected by the environment, etc.	Surfactant selection, ratio of oil phase to water phase, reaction temperature, reaction time, stability regulator, etc.	[30,31,41,49]
microwave method	High efficiency and energy saving, high product quality, easy operation, wide application range, etc.	It is difficult to control reaction conditions, high equipment costs, and limited selection of reaction systems, etc.	Microwave power, reaction time, reaction temperature, reaction solvent, reaction container, etc.	[43,45]

**Table 2:** Improvement of mechanical properties [8].

CaSO_4_ Content/wt.%	Curing Time/d	Flexural Strength Improvement/%	Compressive Strength Improvement/%
	3	6.9	2.8
3	7	7.7	3.7
	28	8.4	4.2
	3	14.4	3.2
5	7	22.2	4.1
	28	28.3	8.5
	3	11.3	4.1
7	7	17.5	4.7
	28	21.8	5.6

## Data Availability

Data are contained within the article.
